# Identification of Bacteria and Viruses Associated with Patients with Acute Febrile Illness in Khon Kaen Province, Thailand

**DOI:** 10.3390/v16040630

**Published:** 2024-04-18

**Authors:** Rungrat Jitvaropas, Vorthon Sawaswong, Yong Poovorawan, Nutthanun Auysawasdi, Viboonsak Vuthitanachot, Sirima Wongwairot, Wuttikon Rodkvamtook, Erica Lindroth, Sunchai Payungporn, Piyada Linsuwanon

**Affiliations:** 1 Division of Biochemistry, Department of Preclinical Science, Faculty of Medicine, Thammasat University, Pathum Thani 12120, Thailand; jrungrat@tu.ac.th; 2 Center of Excellence in Systems Microbiology, Faculty of Medicine, Chulalongkorn University, Bangkok 10330, Thailand; vorthon.s@chula.ac.th; 3 Center of Excellence in Clinical Virology, Department of Pediatrics, Faculty of Medicine, Chulalongkorn University, Bangkok 10330, Thailand; yong.p@chula.ac.th; 4 Department of Entomology, US Medical Directorate-Armed Forces Research Institute of Medical Science, Bangkok 10400, Thailand; nutthanuna.ca@afrims.org (N.A.); sirimaw.ca@afrims.org (S.W.); erica.lindroth.mil@afrims.org (E.L.); 5 Chumpare Hospital, Chum Phae, Khon Kaen 40130, Thailand; viboonvut@hotmail.co.th; 6 Analytic Division, Royal Thai Army Component-Armed Forces Research Institute of Medical Science, Bangkok 10400, Thailand; rwuttikon@gmail.com; 7 Department of Biochemistry, Faculty of Medicine, Chulalongkorn University, Bangkok 10330, Thailand

**Keywords:** undifferentiated acute febrile illness, scrub typhus, rickettsiosis, murine typhus, spotted fever rickettsiosis, dengue virus, Guadeloupe mosquito virus, virome, metagenomic next-generation sequencing

## Abstract

The majority of cases of undifferentiated acute febrile illness (AFI) in the tropics have an undefined etiology. In Thailand, AFI accounts for two-thirds of illnesses reported to the Ministry of Public Health. To characterize the bacterial and viral causes of these AFIs, we conducted molecular pathogen screening and serological analyses in patients who sought treatment in Chum Phae Hospital, Khon Kaen province, during the period from 2015 to 2016. Through integrated approaches, we successfully identified the etiology in 25.5% of cases, with dengue virus infection being the most common cause, noted in 17% of the study population, followed by scrub typhus in 3.8% and rickettsioses in 6.8%. Further investigations targeting viruses in patients revealed the presence of Guadeloupe mosquito virus (GMV) in four patients without other pathogen co-infections. The characterization of four complete genome sequences of GMV amplified from AFI patients showed a 93–97% nucleotide sequence identity with GMV previously reported in mosquitoes. Nucleotide substitutions resulted in amino acid differences between GMV amplified from AFI patients and mosquitoes, observed in 37 positions. However, these changes had undergone purifying selection pressure and potentially had a minimal impact on protein function. Our study suggests that the GMV strains identified in the AFI patients are relatively similar to those previously reported in mosquitoes, highlighting their potential role associated with febrile illness.

## 1. Introduction

Undifferentiated AFI, characterized by a high fever persisting for at least three days and lacking a readily identifiable cause, is a leading cause of medical consultations and hospitalization globally, including in Thailand. Its broad definition encompasses a diverse range of potential etiologies, posing a diagnostic challenge that can impede timely and effective treatment, particularly in resource-limited settings or areas with a constrained diagnostic laboratory capacity.

In Thailand, the identification of causality associated with AFI has focused primarily on specific bacterial, viral, and parasitic pathogens due to their notification requirements to the Ministry of Public Health of Thailand (Thai-MoPH). However, this focus overlooks a significant portion of the etiological landscape. Multiple pathogens that have been reported by other countries in Southeast Asia remain neglected and potentially contribute to undiagnosed AFI cases in Thailand [1]. This limitation hinders the development of appropriate public health interventions, ultimately perpetuating the disease burden. Misdiagnosing a viral infection as bacterial can lead to several negative consequences, including the overuse and misuse of antibiotics and a subsequent increase in antibiotic-resistant bacteria. Accurate diagnosis, evidence-based data, and the epidemiology of public-health-significant pathogens are valuable tools for guiding clinicians in endemic areas when managing AFI cases.

The detection of viral and bacterial pathogens causing AFI varies significantly depending on the detection technique used and the location of the study. Data from the Thai-MoPH suggests that dengue virus (DENV) is the most common cause of AFI, with an annual incidence level of 181.29:100,000 reported in 2023 [2]. DENV infection is present throughout the year but peaks during the rainy season, which begins in July and continues until October in Thailand. One issue that needs to be considered is that some DENV-positive cases reported in the database may rely solely on a physical examination and may not be confirmed by a laboratory diagnostic test. The true burden of DENV infection and the distribution of its serotypes remain largely unknown [3,4]. This significantly hinders strategic planning for effective population-level vaccine interventions. Unlike DENV, which occurs nationwide, cases of malaria are concentrated in border regions, where its mosquito vector is abundant [5,6,7]. Fortunately, the overall incidence and public health impact of malaria are declining, with a promising vaccine expected in the near future [8].

Scrub typhus group (STG), caused by the highly diverse *Orientia tsutsugamushi* infection, is another endemic disease in Thailand. Competent vectors, trombiculid mites, are found throughout the country and are more abundant during periods of increased ambient temperature, humidity, and rainfall [9,10,11,12]. Although the disease is reported in all regions, the hotspots are concentrated in the northern and southern regions, with peak prevalence from the late rainy season to early winter, coinciding with the peak season of DENV [2,13]. Although STG is treatable, the disease poses a diagnostic challenge due to the low prevalence of eschar lesions (necrotic wounds at the chigger bite site), and its symptoms are indistinguishable from those of other tropical diseases. A misdiagnosis of STG can result in inappropriate or delayed treatment, potentially increasing the fatality rate, which can reach up to 30% in some settings [14]. The incidence level of STG in Thailand demonstrated a notable increase between 2022 and 2023, with rates rising from 9.41:100,000 to 12.88:100,000 [2], accounting for a 36.9% increase in the incidence. This significant rise coincides with the current trends reported in multiple countries within the Tsutsugamushi Triangle, indicating a worrying surge in STG cases [15,16,17,18]. This trend, along with the urbanization of the disease and the identification of STG cases outside of the traditionally endemic regions [19,20,21,22], has heightened concerns regarding STG as a re-emerging threat. It also warrants further investigation into potential contributing factors and necessitates effective public health interventions to mitigate the growing burden of STG.

Spotted fever group rickettsiosis (SFG) and murine typhus group (TG) are prevalent in Southeast Asian countries and are recognized as the second most common ectoparasite-borne diseases after STG [23,24,25]. Several *Rickettsia* species, such as *Rickettsia honei* and *R. felis*, cause SFG and are transmitted to humans through infectious tick bites. Additionally, trombiculid mites are suspected to be potential vectors [26,27,28]. TG, however, is caused by typhus group *R. typhi* and is transmitted to humans through contact with the infected feces of rodent fleas. Despite their significant contribution to AFI cases in many neighboring countries, it is not mandatory to report SFG or TG to the Thai MoPH. The degree to which SFG and TG are under-identified or reported and the incidence of confounding with cases such as DENV, STG, and other tropical diseases remain largely unknown.

Metagenomic next-generation sequencing (mNGS) has been considered a transformative approach for pathogen detection, and it can effectively surpass traditional molecular techniques in both sensitivity and specificity, as well as cost-effectiveness [29]. Its high-throughput capabilities have been used to support several applications in human research, for example, in enhancing microbiome analyses to identify antimicrobial-resistant bacteria in patients or hospital settings [30], identifying emerging or re-emerging pathogens [31,32,33], forensic investigations [34], and cancer diagnosis [29,35]. Additionally, mNGS enables the rapid and direct detection of unknown infectious agents in clinical samples, leading to continuous improvements in diagnostic accuracy [35]. This potential has been realized through the discovery of several novel viruses and bacteria using mNGS technology [36,37,38,39,40].

Given the diverse etiological landscape of AFI, our study aims to fill the knowledge gaps in identifying bacterial and viral pathogens targeting ectoparasite-borne and mosquito-borne diseases using targeted PCR-based and serological assays. This focus not only addresses the critical need for a broader understanding of AFI etiologies but also underscores the importance of including a wider array of pathogens in diagnostic evaluations, particularly those that have historically been overlooked or underreported in the healthcare system. Through this approach, our research endeavors to enhance the detection and management of AFI to pave the way for improved public health strategies and interventions in Thailand and potentially other regions with similar epidemiological profiles. Furthermore, our study leverages mNGS to investigate potential neglected viruses associated with AFI using serum samples that tested negative via traditional PCR methods. We successfully sequenced the complete genomes of four strains of Guadeloupe mosquito virus (GMV), which have previously only been reported in mosquitoes, from AFI patients. This finding suggests the potentially unrecognized role of GMV in humans and expands the spectrum of viral pathogens potentially contributing to AFI.

## 2. Materials and Methods

### 2.1. Study Population and Ethical Considerations

**Figure 1:**
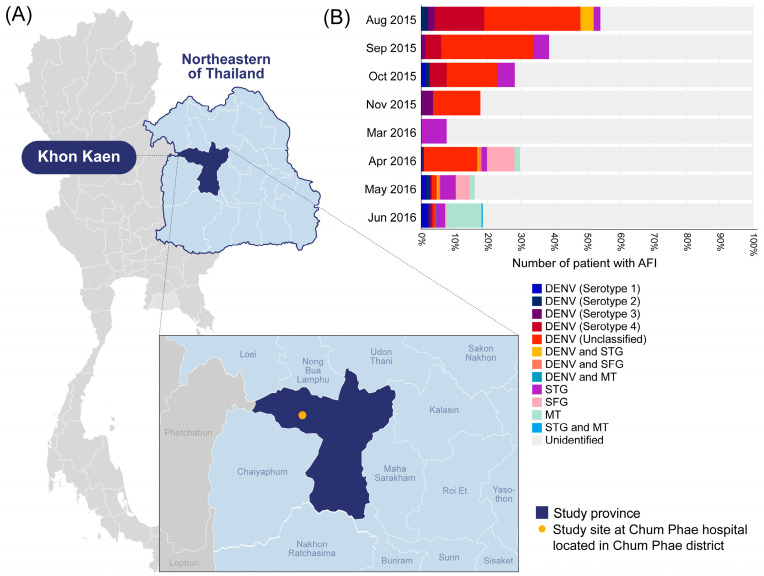
(**A**) Map of Thailand indicating the location of Chum Phae Hospital in Khon Kaen province and (**B**) the infection prevalence of target pathogens identified in our study. Each color on the bar graph represents the pathogens identified in this study, while the areas highlighted in light gray indicate the samples that tested negative for all target pathogens (unidentified). DENV; Dengue virus infection, STG; Scrub typhus, SFG; Spotted fever group rickettsiosis, TG; Murine typhus.

This study was conducted at Chum Phae Hospital, a 200-bed provincial hospital in Khon Kaen province that covers residents in Khon Kaen and nearby Chaiyaphum, Loei, and Nong Bua Lam Phu provinces due to its vicinity (within a 40 km radius). This hospital thus serves as the main healthcare facility in this region (Figure 1). Ethical clearance was granted by the Institutional Review Board of the Faculty of Medicine of Chulalongkorn University and the Walter Reed Army Institute of Research-Human Protection Branch.

To identify etiologies associated with AFI in Khon Kaen province, we used a representative set of residual blood and serum samples provided by Chum Phae Hospital for molecular and serological analyses. This sample set was collected from AFI patients during August 2015 to June 2016. Whole blood and serum samples were collected from AFI patients during their hospital visits, regardless of age or gender, who sought either outpatient or inpatient medical care at Chum Phae Hospital. Patients were included if they fulfilled four primary criteria: (a) no severe underlying disease or immunocompromising treatments, (b) documented acute fever with a tympanic temperature exceeding 37.5 °C upon admission, (c) a history of fever lasting more than two days without a specific focus on pathogens after medical examinations, and (d) documented AFI but a tympanic temperature below 37.5 °C due to self-medication with a fever-reducing drug prior to the hospital visit. AFI patients who reported taking antibiotics were excluded. Samples from patients documented to have an identifiable source of fever, including, but not limited to, urinary tract infection, wound infection, sinusitis, bronchitis, or pneumonia, were also excluded.

The samples were subjected to hemoconcentration tests, including tests of white blood cell count, platelet count, hematocrit, and albumin or specific pathogens of interest based on clinical necessity or guidance from local clinicians. After the completion of the general blood examinations, the de-identified residual blood and serum samples were transferred into two new sterile tubes (two tubes/type) and then stored at −20 °C before being sent to the Center of Excellence in Clinical Virology, Chulalongkorn University and Department of Entomology, WRAIR-AFRIMS, in Bangkok. The samples were subsequently stored at −80 °C until analysis. The samples were sent within one week after collection on average.

### 2.2. Screening for Viral and Bacterial Pathogens

The blood and serum samples from individual patients were initially screened for the presence of pathogens that have been reported to be endemic in Thailand or neighboring countries using a combination of PCR and/or serological analyses. Screening was conducted for five pathogens including the viral pathogens DENV, Chikungunya virus, and Zika virus, as well as the bacterial pathogen *Orientia tsutsugamushi* (Otsu-causative agent of STG) and the rickettsial agents of SFG and TG. A list of the methodologies used in this study is presented in Table 1. The study area is considered to be a non-malaria area, as fewer than ten cases have been reported over the last ten years [2]. Therefore, malaria screening was not included.

Total nucleic acid was extracted from the blood and serum samples using an IndiMag Pathogen Kit following the manufacturer’s instructions (Indical Biodesign, Leipzig, Germany). Nucleic acid was diluted in 200 µL of an EB elution buffer and subsequently concentrated using a vacuum concentrator (Concentrator plus, Eppendorf, Germany). The final volume of each sample was adjusted to 100 µL using the EB elution buffer. The RNA was reverse-transcribed and synthesized into double-stranded complementary DNA (cDNA) using an iScript™ cDNA synthesis kit (Bio-Rad Laboratories, Hercules, CA, USA) according to the manufacturer’s protocol. If samples showed a detection signal for the pathogen of interest, the sequencing of specific gene(s) of the positive pathogens was performed to achieve nucleotide sequences for genetic diversity and further characterization (Table 1). Amplified PCR products were purified using a MinElute PCR purification kit (Qiagen, Hilden, Germany) and were subsequently sent for bidirectional nucleotide sequencing (1st BASE company, Singapore).

### 2.3. Viral Nucleic Acid Extraction and cDNA Synthesis for mNGS

A total of 87 serum samples representative of each patient, regardless of patient clinical presentation or demographic information, were pooled at an equal amount of 100 µL per sample, with 9 or 10 samples per pool, yielding a total of 9 pooled samples for library preparation (samples VF1 to VF9). The pooled serum samples were subjected to virion enrichment following a previously published protocol with some modifications [45]. In brief, each pooled serum sample was filtered through a 0.45 µm syringe filter (Sartorius, Göttingen, Germany), and 500 µL of the filtrate was treated with nuclease to eliminate cell-free, host genetic materials and non-encapsulated nucleic acid. A mixture of nuclease containing 30U of RNase One (Promega, Madison, WI, USA), 3 U of Baseline-ZERO (Epicentre, Madison, WI, USA), 30 U of Benzonase (Novagen, Darmstadt, Germany), and 14 U of Turbo DNase (Thermo Fisher Scientific, San Francisco, CA, USA) in 1× Turbo DNase buffer was added to the filtrate. The reaction was incubated at 37 °C for 1.5 h, and the nucleic acid was extracted using a MagMAXTM viral RNA isolation kit (Thermo Fisher Scientific, San Francisco, CA, USA) following the manufacturer’s protocol. First-strand cDNA synthesis was carried out using a RevertAid cDNA synthesis kit (Thermo Fisher Scientific, San Francisco, CA, USA) with 100 pmol of tailed random oligo-nanomers (5′-GTT TCC CAC TGG AGG ATA NNN NNN NNN-3′) in a 20 µL reaction as per the standard protocol. For second-strand cDNA synthesis, the reaction was mixed with 5 U Klenow Fragment DNA polymerase (New England Biolabs, Ipswich, MA, USA) and incubated at 37 °C for 60 min, followed by incubation at 75 °C for 20 min.

### 2.4. Library Preparation for mNGS Analysis

The cDNA of viral RNA was synthesized using a sequence-independent, single-primer amplification method. The PCR reaction contained 2 µM of primer (5′-GTT TCC CAC TGG AGG ATA-3′), 0.2 U of Phusion DNA polymerase (Thermo Fisher Scientific, San Francisco, CA, USA), 0.25 of mM dNTPs, 5 µL of cDNA template, and 1× Phusion HF buffer. The thermal profile was 95 °C for 5 min; 5 cycles of 95 °C for 30 s, 59 °C for 60 s, and 72 °C for 90 s; 35 cycles of 95 °C for 30 s, 59 °C for 30 s, and 72 °C for 90 s (+2 s per cycle); followed by 72 °C for 10 min and holding at 4 °C. The PCR products were then fragmented using an M220 Covaris-focused ultrasonicator (Covaris, Woburn, MA, USA) with a target fragment size of 400 bp. The fragmented DNA was used for DNA library preparation using an NEBNext Ultra II DNA Library Prep Kit for Illumina (New England Biolabs, Ipswich, MA, USA). Finally, the library was paired-end-sequenced (2 × 250 cycles) using the Illumina MiSeq sequencing platform with a 5% PhiX spike-in.

### 2.5. Bioinformatics and Virus Identification

The quality control of FASTQ sequences was performed using FastQC [46]. Adaptor sequences and low-quality sequence reads with a q-score lower than Q20 were removed using Trimmomatic v0.36 [47]. Host reads were eliminated by mapping the remaining sequences to the human reference genome (GRCh38) using Bowtie2 [48]. Unmapped reads were assembled de novo into contigs using EnsembleAssembler [49]. These contigs were then BLASTx-searched against the NCBI viral protein (vp) database (https://ftp.ncbi.nlm.nih.gov/refseq/release/viral/, accessed on 1 December 2020) with an e-value cutoff of 1 × 10^−10^. Taxonomically classified contigs were subsequently used as reference sequences for identifying viral reads in the experimental datasets and counting hits per viral taxon through Bowtie2 mapping with default parameters. Subsequently, contigs matching eukaryotic viruses were further subjected to a BLASTx analysis against the NCBI non-redundant protein sequences (nr) database to verify and exclude false-positive hits. The BLAST hit result with the lowest e-value in the vp and nr searches was used for definitive taxonomic classification. Genome acquisitions of interesting viral contigs were performed using Geneious Prime v2021.1. Briefly, contigs were iteratively mapped using Geneious mapper with five replicates. Consensus genomes were constructed and annotated using an open reading frame (ORF) predictor within Geneious Prime v2021.1 to validate the translation frame and elucidate the genomic structure of the viruses.

### 2.6. PCR Detection of GMV in Individual Serum Samples

Until recently, no standard gene existed for the genotypic characterization of GMV. Based on the genome analysis of the GMV sequences retrieved from the GenBank database, the RNA-dependent RNA polymerase (RdRp) gene displayed a high nucleotide variation, which is a desirable characteristic for distinguishing species and strains in various viruses. Therefore, we utilized the RdRp gene to screen for the presence of GMV in the AFI patient samples. Specific primers for nested PCR were designed based on the GMV sequence obtained from GenBank, including GMV (accession no. MN053803.1) and Renna virus (accession no. MK285337.1) (Table S1). The PCR mixture included 0.2 μM of each primer, 0.2 mM of dNTPs, 0.625U of Taq DNA Polymerase (New England Biolabs, Ipswich, MA, USA), 0.2 μM of 1× Standard Taq Reaction Buffer, and 1 μL of cDNA template prepared from serum samples and nuclease-free water to achieve a final volume of 25 μL. The thermal cycling conditions were performed as follows: initial denaturation at 95 °C for 30 s, followed by 40 cycles of 95 °C for 15 s, 58 °C for 30 s, and 68 °C for 25 s, with a final extension at 68 °C for 5 min and holding at 4 °C. The first PCR product was used as the DNA template, and the same condition was applied for the second round of PCR. The quality and integrity of the cDNA in each sample were evaluated through the amplification of the housekeeping gene encoding for human glyceraldehyde 3-phosphate dehydrogenase following a previously described protocol [50]. Amplicons of the expected size were visualized via agarose gel electrophoresis and verified through Sanger sequencing.

### 2.7. Identification of the Complete GMV Genome

The complete genomes of GMV were amplified and sequenced from four serum samples obtained from two AFI patients in 2015 (S90 and S247) and two dengue-suspected patients in 2019 (D256 and D341) using semi-nested PCR approaches. Specific primer sets were designed based on the conserved regions shared between the Renna virus and 17 GMV reference sequences from the GenBank database (Table S2). The primer specificity was verified through an in silico analysis using the NCBI Primer-BLAST tool (https://www.ncbi.nlm.nih.gov/tools/primer-blast/, accessed on 20 May 2021). Each 25 μL PCR reaction mixture contained 0.2 μM of each primer, 0.2 mM of dNTPs, 0.25 μL of Phusion™ Plus DNA Polymerase (Thermo Fisher Scientific, San Francisco, CA, USA), 1× Phusion™ Plus Buffer, and 1–5 μL of the cDNA or 1^st^ PCR product template. The PCR cycling conditions were as follows: denaturation at 98 °C for 30 s, followed by 35–40 cycles of 98 °C for 10 s, 55 °C for 10 s, 72 °C for 30 s, and a final extension at 72 °C for 5 min. The second round of PCR was performed similarly using the initial PCR product as the template. The PCR products were purified using a QIAquick Gel Extraction Kit or a QIAquick PCR Purification Kit (Qiagen, Hilden, Germany) and subsequently bidirectionally sequenced in duplicate reactions.

### 2.8. Sequence Analysis and Phylogenetic Tree Construction

Nucleotide sequences were verified for peak quality and then assembled using overlapping sequences utilizing BioEdit software v.7.2 [51]. Subsequently, the whole-genome sequences were characterized through a BLAST analysis and verified for quality by predicting in-frame ORFs and comparing them with other GMV sequences. Multiple whole-genome sequences were then aligned with the consensus sequences using the Clustal W algorithm [52]. ORF prediction for the complete genomes was performed using the NCBI ORF Finder (https://www.ncbi.nlm.nih.gov/orffinder/, accessed on 18 February 2022) and compared with the available GMV reference sequences for shared structural domains. The full-length nucleotide sequences and each segment were used to infer evolutionary relationships between the patient-amplified viruses and reference strains. Phylogenetic trees were generated using the maximum likelihood method with a General Time Reversible model and 1000 bootstrap replicates in MEGA software v.11 [53].

### 2.9. Statistical Analysis

Various clinical information and laboratory results were used for data analyses, including blood cell counts, biochemical markers, serologic values, and patient demographic information. However, the presence of eschars was not systematically recorded because they are not routinely assessed. A statistical analysis was performed using GraphPad Prism v9.0. This included descriptive statistics, such as frequency distributions and percentages, along with an inferential analysis. In addition, in order to compare the prevalence of DENV infections identified through different methods, a Venn diagram was created using R software v4.3.3 to visualize the overlap of positive results between the different detection methods.

## 3. Results

### 3.1. Etiological Agents Associated with AFI in Khon Kaen

During the study period, 1089 patients visiting Chum Phae Hospital met the study inclusion criteria (Table 2). However, 89 cases were excluded due to incomplete information on fever duration, pre-existing conditions such as cancer or an immunocompromised status, fever from identifiable sources, or self-medication with antibiotics. Therefore, a total of 1000 cases were subjected to further pathogen investigations. The sample selection flowchart is revealed in Figure S1. A nearly equal sex distribution was observed, with 53.7% of the AFI patients being male. The median age was 10 years (interquartile ranges at the 25th and 75th percentiles (IQR): 5–26 years). Over 66% of the study population comprised young children and adolescents. The most frequently reported symptoms, without attributing them to a specific cause, included a high fever, headache, fatigue, cough, and vomiting.

#### 3.1.1. Arboviruses

**Figure 2:**
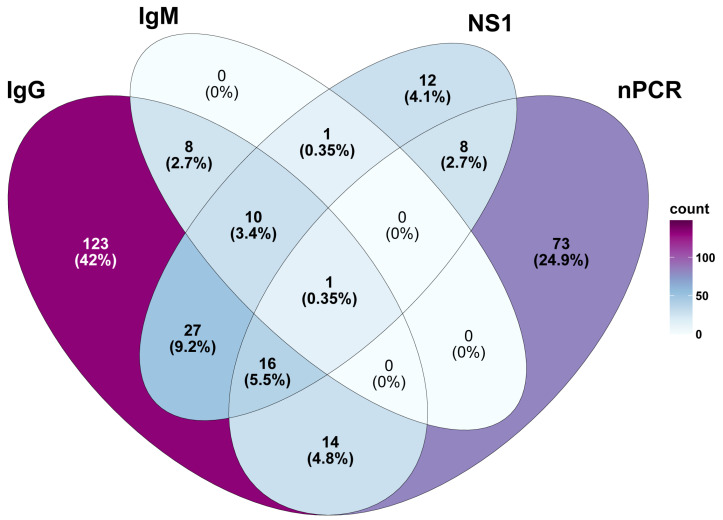
Comparative prevalence of DENV infection in 170 cases identified through various detection methods. Results of ELISA for specific DENV IgG alone are excluded from the analysis to prevent overestimation of the burden of DENV infection in AFI patients.

DENV was the most prevalent pathogen identified among the AFI patients. The PCR-based detection yielded a higher number of positive results than the antigen-based ELISA detection (nonstructural protein 1-based ELISA or NS1-ELISA) (112 cases vs. 75 cases). Only 25 samples were confirmed to be positive or equivocal for DENV through nPCR and NS1-ELISA, suggesting that PCR may be more sensitive for detecting recent infections. Testing for DENV-specific immunoglobulin M (IgM_DENV_) and IgG (IgG_DENV_) was performed using Euroimmun ELISA kits (PerkinElmer Company, Lübeck, Germany). The results revealed that 2% (20/1000) of the AFI patients were positive for IgM_DENV,_ and, of these, 19 patients had developed both IgM_DENV_ and IgG_DENV_. This could indicate a recent infection, as well as the potential for the re-infection of DENV (Figure 2). A significantly higher proportion of the study population developed IgG_DENV_ only, which accounted for 18% (180/1000) of the seroprevalence (Figure 2). This high level of IgG_DENV_ prevalence could be due to several factors, including past DENV infection and endemic exposure in the populations. Given the unavailability of a geometric mean titer of IgG_DENV_ and specific criteria for data interpretation, we opted to exclude IgG_DENV_ data from our final analysis to avoid biases arising from potential past infections or the underlying serological profiles of the patients. Therefore, based on our findings, we estimated that 17% (170/1000) of the AFI patients were actively infected with DENV.

The majority of the DENV cases were school-age children aged 6–12 years (34.7% (59/170)), followed by adolescents (17.1% (29/170)), with a median age of 10 years (IQR: 6–19 years). Before the hospital visit, the patients with DENV infection had an average fever duration of 3 days (IQR: 2–4 days) and an average tympanic temperature of 38.5 °C (IQR: 37.9–39 °C). As shown in Figure 1, most DENV-positive cases were detected during the rainy season and early winter months. A phylogenetic analysis of successfully amplified NS1 gene sequences (53 sequences) revealed DENV serotype 4 as the most dominant serotype during the study period, followed by DENV serotype 2 (30%), DENV serotype 3 (20%), and DENV serotype 1 (10%). When comparing the patients with confirmed ST, SFR, and DENV, the mean platelet (237,541.7 vs. 284,044.1 vs. 219,110.6 cells/uL) and white blood cell (8541.7 vs. 10,279.4 vs. 6771.7 cells/uL) counts of the DENV patients were lower, and the patients were likely to have a high fever (94.7%), rash (4.2%), and fatigue (2.1%). All patients tested negative for Chikungunya virus and Zika virus infections.

#### 3.1.2. *Orientia tsutsugmaushi*

Otsu was detected in 16 patients, accounting for 1.6% of the cases. Our study used a whole-cell antigen immunofluorescence staining assay (IFA) on acute-phase serum samples to test for specific IgM and IgG antibodies against four genogroups of Otsu: Karp, Kato, Gilliam, and TA763. Our findings revealed that 2.1% (21/1000) of the AFI patients had specific IgM antibodies to Otsu (IgM_STG_), with the titer exceeding 1:400, indicating an active Otsu infection status. Conversely, serological assessments of IgG_STG_ in these samples suggested previous exposure in 1.7% of the cases. When comparing the PCR and IgM IFA results for Otsu detection, only one patient tested positive in both assays. In conclusion, 3.8% (38/1000) of the total STG cases were among the AFI patients. No specific clinical characteristics that could distinguish individuals with STG and SFG infection from those with DENV infection were observed.

The median age of the patients with Otsu infection was 25.4 years (IQR: 5.8–47), with a female–male ratio of 1.3:1. Indistinguishable symptoms between STG and other diseases were observed, in which the majority of patients exhibited a high fever (50%), cough (18.8%), and headache (9.4%). Compared to the DENV patients, the STG patients sought medical care later after symptom onset (3 days (IQR: 2–4)). Patients with IgG positive had a median duration of 3 days (IQR: 2.8–4) after symptom onset before the hospital visit. In contrast to the DENV cases, which were detected throughout almost every month of the study period, the STG cases were more predominantly concentrated during the rainy and winter months. The STG patients had a median age of 19 years (IQR: 14–56). Our finding also revealed that the infection prevalence increased similarly with age and gender, as the majority of patients were female. Four STG patients were confirmed to have DENV co-infection with a high fever, and hemorrhagic rash was a common clinical manifestation observed among these patients. We successfully amplified and sequenced 14 nucleotide sequences of the 56 kDa type-specific surface antigen gene of Otsu. A phylogenetic analysis of these sequences revealed that Karp was the most common genogroup among the AFI patients during the study period. However, as our study used pooled whole-cell antigens from Otsu-Karp, Kato, and Gilliam genogroups in the serological assay, we could not determine the levels of multiple infections or cross-reactivity among the STG-seropositive patients.

#### 3.1.3. Rickettsial Infection

The qPCR testing for the presence of rickettsial pathogens yielded a 3.1% (31/1000) infection prevalence among the AFI patients. Rickettsia-positive cases could be found throughout the year, most commonly occurring from April to June, coinciding with the summer and rainy months. The seasonal activity of these diseases contrasts with that of the diseases observed in northern Thailand, where their activity peaks during the late rainy and early winter months. One patient was infected with *Rickettsia* and DENV at the same time. When considering a specific immune response, the antibody prevalence of TG was significantly higher than that of SFG, with 23 AFI patients exhibiting specific IgM_TG_, 14 exhibiting both specific IgM_TG_ and IgG_TG_, and 1 exhibiting specific IgG_TG_ against *R. typhi* at a dilution titer of 1:200. None of the tested AFI patients had specific antibodies against *R. honei,* implying the possibility of a lower risk of local SFG transmission in this area. There were no significant differences in the exposure of the study age distributions between STG and TG (*p*-value = 0.0801), and STG IgG-seropositive patients were more likely to be adults aged 30 years, while TG patients were aged 19.3 years. In addition to a high fever, the TG patients reported febrile illness (8.3%), cough (8.3%), itching (5.5%), and vomiting (5.5%).

### 3.2. Virome Analysis

**Figure 3:**
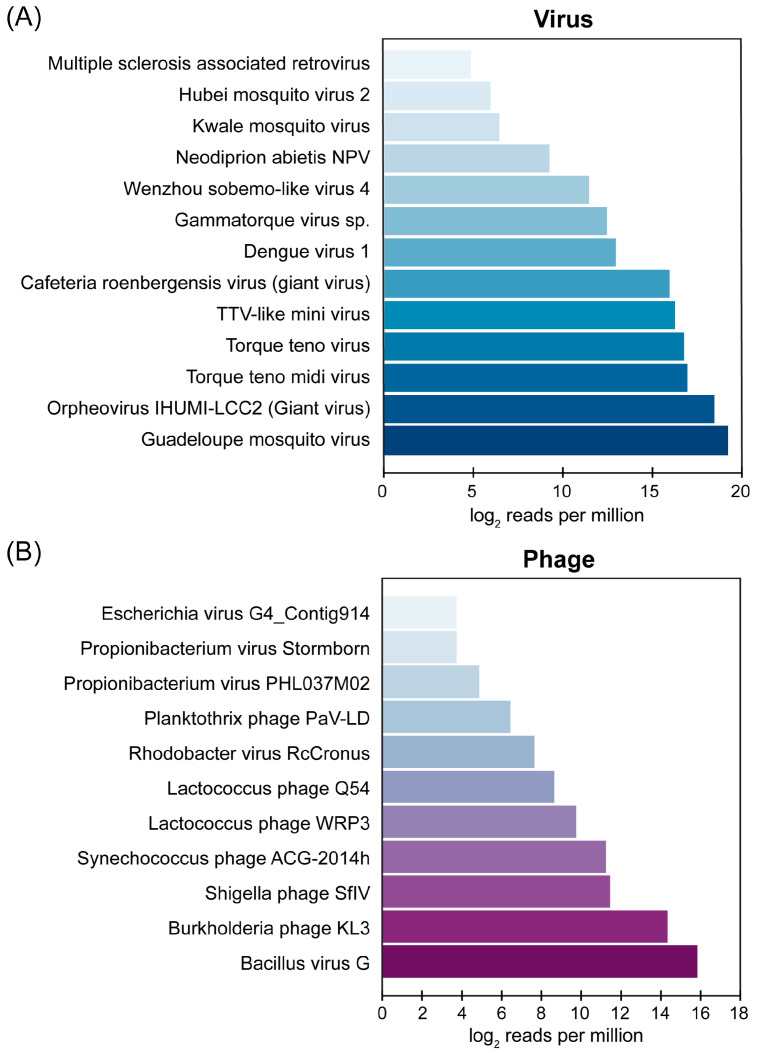
Relative abundance of (**A**) viral species and (**B**) phages identified in pooled AFI serum samples. GMV reads include the reads mapped with contigs hitting GMV and Renna virus (its close relative) because these contigs belong to the same genome of GMV that we recovered and characterized in this study.

To further investigate the presence of neglected viruses in AFI patients, a representative 3.5% (26/734) of the serum samples that were confirmed to be negative for all target pathogens via PCR-based pathogen detection assays were analyzed using mNGS. An additional 61 samples collected from patients who documented a strong clinical suspicion of dengue fever but tested negative for pathogens in screening using NS1 PCR were also included in the analysis. This sample set was collected from patients who visited Chum Phae Hospital in 2019. Therefore, a total of 87 serum samples were used for the mNGS analysis. An analysis of nine pooled samples generated a total of 6.76 million raw reads, with an average of 0.75 million raw reads per pooled sample. After quality filtering, assembled contigs were queried against a viral sequence database using BLASTx. The analysis revealed a diverse virome composition in the AFI serum pools, with known viruses comprising 87.2% (872,193 hits per million reads) and phages contributing 12.8% (127,807 hits per million reads). The percentage of human reads sequenced is reported in Table S3 to assess virion enrichment efficiency. Details on individual contigs, their reference sequence similarity, and normalized read counts are also provided in Table S4. Among the detected viruses, Guadeloupe mosquito virus (GMV), Orpheovirus IHUMI-LCC2, and Torque teno midi virus were the most abundant, while Bacillus virus G and Burkholderia phage KL3 emerged as the dominant phages. The relative species abundances of the AFI serum viromes and phages are illustrated in Figure 3A and 3B, respectively.

### 3.3. Complete Genome Characterization of GMV in Thai Patients

GMV was the dominant viral presence in the mNGS analysis, accounting for 39% of all detected viral reads. While previously identified in mosquitoes in 2019, this marks the first report of GMV presence in AFI patient serum samples. Intrigued by this finding, we further investigated its occurrence by focusing on individual serum samples and determining its infection prevalence. Using nested PCR with GMV-specific primers targeting the RdRp gene, we confirmed the presence of GMV in 4 out of 87 serum samples, representing an infection prevalence of 4.6% in this cohort study. A subsequent sequence analysis of the RdRp genes revealed a high degree of similarity, with a 96% nucleotide identity with both the GMV strain Ab-AAM-5 (MN053793) and the Renna virus clone RENV_S1_Mex_2016 (MK285337) based on the BLASTn analysis and phylogenetic analysis. Among the four GMV-positive patients, two patients presented with AFI and were hospitalized in 2015. The other two patients visited the hospital in 2019 and presented with dengue-like symptoms but tested negative for dengue virus by conventional PCR method and NS1-ELISA. The information of four GMV-positive patients is represented in Table S5.

### 3.4. Complete Genome Characterization of GMV in Thai Patients

**Figure 4:**
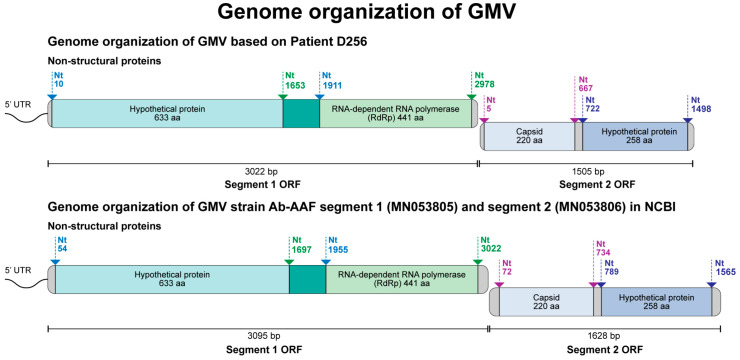
Genome organization of Guadeloupe mosquito virus (GMV) identified in clinical samples in Thailand.

To complete the sequences, we employed primer walking specifically designed from the acquired nucleotide sequences and successfully obtained the complete genome sequences of GMV from all four positive serum samples (S90, S24, D256, and D341). The sequences were deposited in GenBank under the accession numbers OR670427-OR670434. As shown in Figure 4, the GMV viral genome comprises two segments, of which segment 1 (3013–3022 bp) encodes a hypothetical protein and RdRp, and segment 2 (1503–1508) encodes for the capsid protein and another hypothetical protein. The BLASTn results demonstrate that the GMV strains identified in the AFI patients in Thailand are highly similar to the GMV strains Ab-AAF segment 1 (MN053805) and segment 2 (MN053806), with a 93–97% nucleotide identity. The strain Ab-AAF was first isolated from *Aedes aegypti* mosquitoes in Guadeloupe, France, between 2016 and 2017 [54].

### 3.5. Amino Acid Variation in the GMV Genome

An analysis of the deduced amino acid sequences of the GMV genomes revealed several variations between the Thai patient-derived sequences and the reference strain Ab-AAF. These variations primarily occurred in the hypothetical proteins of both segments: in segment 1, they occurred in the positions 84, 87, 102, 114, 123, 134, 135, 140, 170, 398, 408, 538, 540, 541, 543, and 548, and, in segment 2, they occurred in the positions 14, 19, 23, 28, 92, 110, 129, 145, 192, 230, 239, 244, 247, and 248 (Table 3). In contrast, the RdRp gene in segment 1 (positions 162, 312, 346, and 347) and the capsid region in segment 2 (positions 87, 107, and 210) displayed fewer variations. Overall, the amino acid sequences within each coding region were highly conserved among the four Thai patient isolates (Table 3).

### 3.6. Phylogenetic Analysis of GMV Complete Genome Sequences

**Figure 5:**
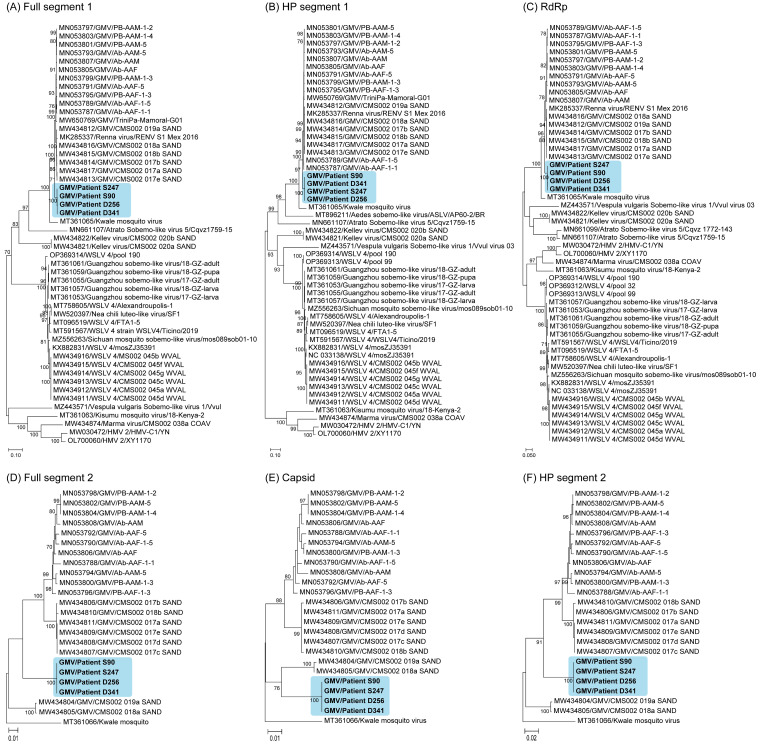
Phylogenetic analysis of GMV and other viruses based on nucleotide sequences. The phylogenetic tree was constructed using the maximum likelihood method, a General Time Reversible model with bootstrap replications (*n* = 1000). Phylogenetic tree of the full-length sequences of segment 1 of GMV ((**A**); full segment 1), the hypothetical protein ((**B**); HP segment 1), and the RdRp-encoding region ((**C**); RdRp) in segment 1 and the full-length sequences of segment 2 ((**D**); full segment 2), the capsid-encoding segment ((**E**); capsid), and the hypothetical protein ((**F**); HP segment 2) in segment 2.

A nucleotide sequence analysis revealed a high similarity among the GMV strains identified in the Thai patients, with a 99.6–99.9% identity for segment 1 and a 99.6–100% identity for segment 2. The maximum likelihood phylogenetic trees constructed using complete nucleotide sequences of both segments consistently grouped the four strains of GMV from the Thai patients (Figure 5). This indicates their close evolutionary relationship and suggests that they have potential for continued circulation in the study area. A phylogenetic analysis of the full length of segment 1 showed that GMV from the Thai patient isolates was most closely related to the GMV and Renna virus reference strains (Figure 5A). Similar close relationships were also observed in separate analyses of each gene, including the hypothetical protein of segment 1 (Figure 5B) and RdRp (Figure 5C). An analysis of the full length of segment 2 (Figure 5D), capsid (Figure 5E), and hypothetical protein in segment 2 (Figure 5F) revealed a very close relationship with the mosquito GMV strains. Determining the convergent evolution of the GMV strains in Thailand requires further experimental studies on the functional compatibility of potentially reassorted segments from different viruses and a comprehensive analysis of evolutionary mechanisms.

## 4. Discussion

By utilizing a combination of targeted molecular and serological assays, our study identified the etiology of AFI in 25.5% of cases, with DENV being the most common cause of hospital visits, accounting for 17%. Notably, our findings revealed that rickettsial infections were the second most frequent causality, with a positivity percentage almost twice that of STG (6.8% vs. 3.8%), underscoring the true neglected status of rickettsial diseases in Thailand. Potential limitations that need to be considered are the use of genus-specific primers for rickettsia detection and the limited volume of some positive samples. This limited our ability to perform multi-locus sequence analyses to confirm the species of rickettsia. The actual burden of SFG and TG warrants further investigation. Compared to other studies in Southeast Asian countries examining DENV, STG, and TG in AFI patients, the seroprevalence reported in our study based on a specific IgG response might be considered lower [55,56]. This discrepancy could be due to our inclusion criteria not having specific age restrictions, resulting in a large number of young children and adolescents being included in the study. The unequal age distribution might not accurately reflect the population structure and could bias the results toward pathogens primarily affecting children. In contrast, other studies might have excluded preschool children or used enrollment criteria specifically targeting patient groups that might have ectoparasite-borne pathogen infections.

When analyzing potential viral pathogens among AFI patients previously found to be negative for known pathogens using mNGS, we found that GMV constituted the largest proportion of viral profiles of the pooled samples and subsequently confirmed the presence of GMV in four individual samples, suggesting a minimum positivity rate of 4.5% in the representative sample set. This finding also raises questions about its potential role in human infection. Prior to this study, GMV was only detected in mosquitoes, including *Aedes aegypti* (Guadeloupe, 2016–2017) [54] and *Culex quinquefasciatus* (Brazil, 2022) [57], and it has not previously been reported in humans. The subsequent discovery of GMV in Brazil and China, geographically distant from Guadeloupe, suggests a potential mosquito-borne route of transmission [57,58]. This is further supported by its presence in field-collected *Aedes albopictus* mosquitoes but not in laboratory-reared mosquito colonies in China, suggesting the potential for horizontal acquisition from the environment [58]. In addition to GMV, other mosquito-borne viruses were also detected in the Thai AFI viromes, including Wenzhou sobemo-like virus 4, Hubei mosquito virus-2, and Kawale mosquito virus. In a previous report, Wenzhou sobemo-like virus 4 and Hubei mosquito virus-2 were the most common viruses in Guadeloupe mosquitoes [54]. Due to the lower abundance of these viruses, we did not individually screen samples for their presence in this study. However, future studies with larger sample sizes might be warranted to explore their role in diseases.

The GMV strains identified in the AFI patient serum samples in this study displayed the same genome organization as the reference strain Ab-AAF from mosquitoes. However, segments 1 (3095 bp) and 2 (1628 bp) of the GenBank reference sequence strain Ab-AAF were longer than those of our strains (segment 1: 3013–3022 bp, segment 2: 1503–1508 bp). The coding sequences of all GMV strains analyzed here were identical in length. A phylogenetic analysis clustered the four Thai patient strains together with other GMV strains, indicating their close evolutionary relationship. Importantly, our findings differ from those of previous studies, where GMV isolates from mosquitoes were more closely related to Wenzhou sobemo-like virus-4 and Hubei mosquito virus-2 [54]. In contrast, the Thai strains exhibited a closer relationship with the GMV strains from mosquitoes. This finding suggests previously unknown transmission dynamics or potential zoonotic spillover events, warranting further investigation in regions with endemic mosquito-borne diseases to understand the true epidemiological profile and potential virulence of this virus in humans.

An analysis of the deduced amino acid sequences of the GMV Thai strains revealed 37 amino acid positional differences from the mosquito-borne GMV sequences. These substitutions were consistently observed within the patient-derived sequences. While most substitutions resulted in amino acids of the same class, certain changes, particularly in hypothetical protein-encoding segments 1 and 2, involved transitions from nonpolar to polar residues. Given the limited understanding of GMV gene function, the impact of these substitutions on virulence remains unclear. Future studies comparing viruses from different sources, including propagation in mosquito cell lines or live mosquitoes, followed by animal model inoculation, can shed light on potential pathogenicity variations.

Other viruses with unknown pathogenicity in humans were also detected in this study in varying amounts. Orpheovirus IHUMI-LCC2 is the Giant virus first reported in 2018 in rat stool samples [59]. Recent evidence suggested that Orpheovirus can infect free-living amoeba *Vermamoeba vermiformis* and could be detected in water samples collected from hospital networks [60]. This amoeba was also identified in a painful ulcer near the eye of one patient, suggesting its potential role in infecting humans [61]. Many diverse anelloviruses, including TTV, TTMDV, TTMV, and Gammatorque virus sp. of the family Anelloviridae, were identified in our virome profile. Data from previous studies suggest that this virus family serves as commensal viruses, which could be detected in both healthy individuals and patient groups [62]. Some viruses in this family are frequently reported in humans, especially TTV, TTMDV, and TTMV [63]. Humans acquire adenoviruses in early childhood; however, the source of infection is still unknown [64]. Although information about the role of these viruses in humans is largely unknown, a study reported by Spandole-Dinu, Sonia et al. indicated that TTV, TTMDV, and TTMV were more commonly detected in patients than in healthy individuals [65]. Our virome analysis also revealed sequence matches with *Cafeteria roenbergensis* virus [66] and *Neodiprion abietis* NPV [67]. These viruses are double-stranded DNA viruses that have been reported in marine zooplankton and the balsam fir sawfly, respectively.

In addition to the mosquito-associated viruses, our sequence analysis identified some sequence matches with human viral pathogens, including DENV serotype 1 and MSRV. DENV is a mosquito-borne virus causing an acute febrile illness known as dengue hemorrhagic fever [68]. Although we employed two methods for screening DENV, identifying DENV sequences in the sample pool using mNGS implied that it is a technique with a higher performance than traditional techniques, including PCR and ELISA [29]. MSRV is an enveloped single-stranded positive RNA virus and a member of the human endogenous retrovirus W family. It was initially isolated from patients with multiple sclerosis and demyelinating diseases [69].

Besides human and mosquito viruses, bacteriophages were also detected in the virome profile. This finding is noteworthy, as previous virome studies have not reported the detection of bacteriophages [36,70,71]. In this study, eleven bacteriophages were identified in very small proportions. Bacillus virus G and Burkholderia phage KL3 were the two most dominant bacteriophages. Bacillus virus G is a dsDNA virus belonging to the family *Myoviridae* class *Caudoviricetes* (NCBI:txid2884420). Burkholderia phage KL3 is a myovirus with a genome length of 40,555 base pairs, and it acts against *Burkholderia cenocepacia* CEP511. *Burkholderia cepacia complex* (BCC) is a group of opportunistic bacterial pathogens that cause infections in cystic fibrosis patients [72].

We recognize that our exploratory study has certain limitations. First, we sampled only 10% of the negative samples for a virome analysis. This relatively small sample size could have potentially limited our ability to detect neglected viruses in AFI patients that were not included in the target screening. Although we individually screened samples to determine the prevalence of GMV, our primary focus was on the limited set of samples. This approach may constrain our understanding of the actual prevalence of GMV in a broader populational study. Second, our recent study did not enroll healthy controls from the same study area. Consequently, this limitation hindered our ability to distinguish between commensal virus species and those potentially pathogenic associated with AFI. Moreover, GMV detections in healthy people should be performed in future studies to investigate the viral role of an opportunistic virus. Third, our study did not examine the bacteriome and mycobiome in AFI patients due to the limited volume of plasma samples available for analysis. As a result, we were unable to provide data on bacteria, fungi, or co-infection of the same patients that might have contributed to fever in this cohort. A previous study conducted on Chinese AFI identified various bacterial species, including *Streptococcus* sp. [70]. Finally, a limitation of the Illumina NGS method used in our study is the generation of short-read sequences, which could be enhanced by incorporating long-read nanopore sequencing for a more comprehensive analysis.

## 5. Conclusions

This study investigated the virome of AFI patients in Thailand and identified both known and previously unreported viruses in clinical samples obtained from them. Notably, we also characterized and reported the complete genomes of GMV strains in clinical samples for the first time in human cases. Nevertheless, further studies should investigate the pathogenesis of GMV and confirm that it is a human pathogen. The knowledge gained from this study will benefit medical personnel and decision-makers, contributing to the development of strategic plans for the diagnosis and treatment of AFI patients in Thailand.

## Figures and Tables

**Table 1:** Summary of pathogens initially screened in this study.

Etiological Agent	Method	Detection Criteria	Objective	Limit of Detection (GEC/µL)	References
Dengue virus Chikungunya virus Zika virus	Realtime RT-PCR	Manufacturer’s criteria ZDC Multiplex RT-PCR Assay for detection of Zika virus, dengue virus, and Chikungunya virus	Pathogen detection	1 GEC/µL	Bio-Rad
Dengue virus	IgM and IgG ELISA	Nonstructural protein 1 (NS1)	Serological screening	N/A	Euroimmune
*Orientia tsutsugamushi*	Realtime-PCR and nested PCR	Positive by 2 of 3 qPCR analysis of 47-kDa high-temperature requirement A gene Nucleotide sequence analysis of 56-kDa type-specific antigen gene	Pathogen detection and confirmation	10 GEC/µL	[41,42]
	IgM or IgG IFA	>4-fold rising titer in convalescence sera OR antibody titer > 1:400 in single serum samples	Serological screening	N/A	[43]
*Rickettsia* spp.	Realtime-PCR	Positive by 2 of 3 qPCR analysis of *ompB* gene amplification	Pathogen detection	10 GEC/µL	[44]
	Realtime-PCR and nested PCR	Positive by 2 of 3 qPCR analysis of 17-kDa type-specific antigen gene (17*tsa*) amplification Nucleotide sequence analysis of 17*tsa* gene	Pathogen detection	10 GEC/µL	[44]
	IgM or IgG IFA	>4-fold rising titer in convalescence serum samples OR antibody titer > 1:400 in single serum samples	Serological screening	N/A	[44]

GEC/µL indicates the genomic equivalent copy per microliters of the sample.

**Table 2:** Summary of demographic data of AFI patients.

Criteria	Range or Total	Average	Median (Q1–Q3)
Total tested patients	1000 patients		
Age (range)	0–88 years	18.9 years	10 years (5–26)
Infant (0–1 year)	56 (5.6%)	0.8 years	1 year (0.67–1)
Toddler (>1–3 years)	120 (12%)	2.4 years	2 years (2–3)
Preschool (>3–5 years)	104 (10.4%)	4.54 years	5 years (4–5)
School-age children (>5–12 years)	280 (28%)	8.65 years	9 years (7–10)
Adolescent (>12–18 years)	107 (10.7%)	14.93 years	15 years (14–16)
Young adult (>18–24 years)	74 (7.4%)	21.31 years	21 years (20–23)
Adult (>24–64 years)	213 (21.3%)	42.44 years	42 years (33–52)
Elderly (>64 years)	46 (4.6%)	75.55 years	75.5 years (72–79)
Tympanic temperature (°C)	35.3–40.3 °C	38.1 °C	38.2 °C (37.5–39.0)
Fever duration prior to hospital visit	2–10 days	3.1 days	3 days (2–4)
Male	535 (53.6%)		
Female	465 (46.4%)		

**Table 3:** Amino acid variation among the hypothetical protein and RdRp region in segment 1, as well as the capsid and hypothetical protein region in segment 2 of the GMV genome.

	Variation in Deduced Amino Acid in the Hypothetical Protein of Segment 1 (633 Amino Acids)															
	84	87	102	114	123	134	135	140	170	398	408	538	540	541	543	548
GMV strain Ab-AAF	H	T	I	I	G	N	G	T	E	L	G	R	S	V	S	R
GMV/Patient S90	D	A	V	T	A	S	S	S	D	I	E	K	N	A	N	K
GMV/Patient S247	D	A	V	T	A	S	S	S	D	I	E	K	N	A	N	K
GMV/Patient D256	D	A	V	T	A	S	S	S	D	I	E	K	N	A	N	K
GMV/Patient D341	D	A	V	T	A	S	S	S	D	I	E	K	N	A	N	K
Virus	**Variation in Deduced Amino Acid in the** **RdRp of Segment 1 (441 Amino Acids)**								**Variation in Deduced Amino Acid in the Capsid of Segment 2 (220 Amino Acids)**							
	**162**	**312**	**346**	**347**					**87**	**107**	**210**					
GMV strain Ab-AAF	Q	I	R	Q					T	A	I					
GMV/Patient S90	H	V	K	H					A	V	V					
GMV/Patient S247	H	V	K	H					A	V	V					
GMV/Patient D256	H	V	K	H					A	V	V					
GMV/Patient D341	H	V	K	H					A	V	V					
Virus	**Variation in Deduced Amino Acid in the Hypothetical Protein of Segment 2 (258 Amino Acids)**															
	**14**	**19**	**23**	**28**	**92**	**110**	**129**	**145**	**192**	**230**	**239**	**244**	**247**	**248**		
GMV strain Ab-AAF	V	E	K	Y	V	A	A	V	V	A	Y	S	V	L		
GMV/Patient S90	I	V	Q	F	I	T	S	I	M	V	G	T	I	S		
GMV/Patient S247	I	V	Q	F	I	T	S	I	M	V	G	T	I	S		
GMV/Patient D256	I	V	Q	F	I	T	S	I	M	V	G	T	I	S		
GMV/Patient D341	I	V	Q	F	I	T	S	I	M	V	G	T	I	S		

## Data Availability

The datasets generated during and/or analyzed during the current study are available from the corresponding author upon reasonable request.

## Supplementary Materials

The following supporting information can be downloaded at https://www.mdpi.com/article/10.3390/v16040630/s1, Figure S1: Sample selection flowchart; Table S1: Designed primer sets for GMV detection and complete genome identification; Table S2: Information on the Renna virus and 17 GMV references used for specific primer design in genome sequence study; Table S3: Sequencing read statistics and host filtering; Table S4: The normalized for million reads and BlastX analysis of each contig; Table S5: GMV patient information and hematology lab results.
