# Supplementary material for: Identification of Bacteria and Viruses Associated with Patients with Acute Febrile Illness in Khon Kaen Province, Thailand
Source: Viruses. 2024 Apr 18;16(4):630. doi: 10.3390/v16040630 (PMC11054472; doi:10.3390/v16040630)
Supplement: Supplementary file 1 [file viruses-16-00630-s001.zip › viruses-2915010-supplementary.pdf]

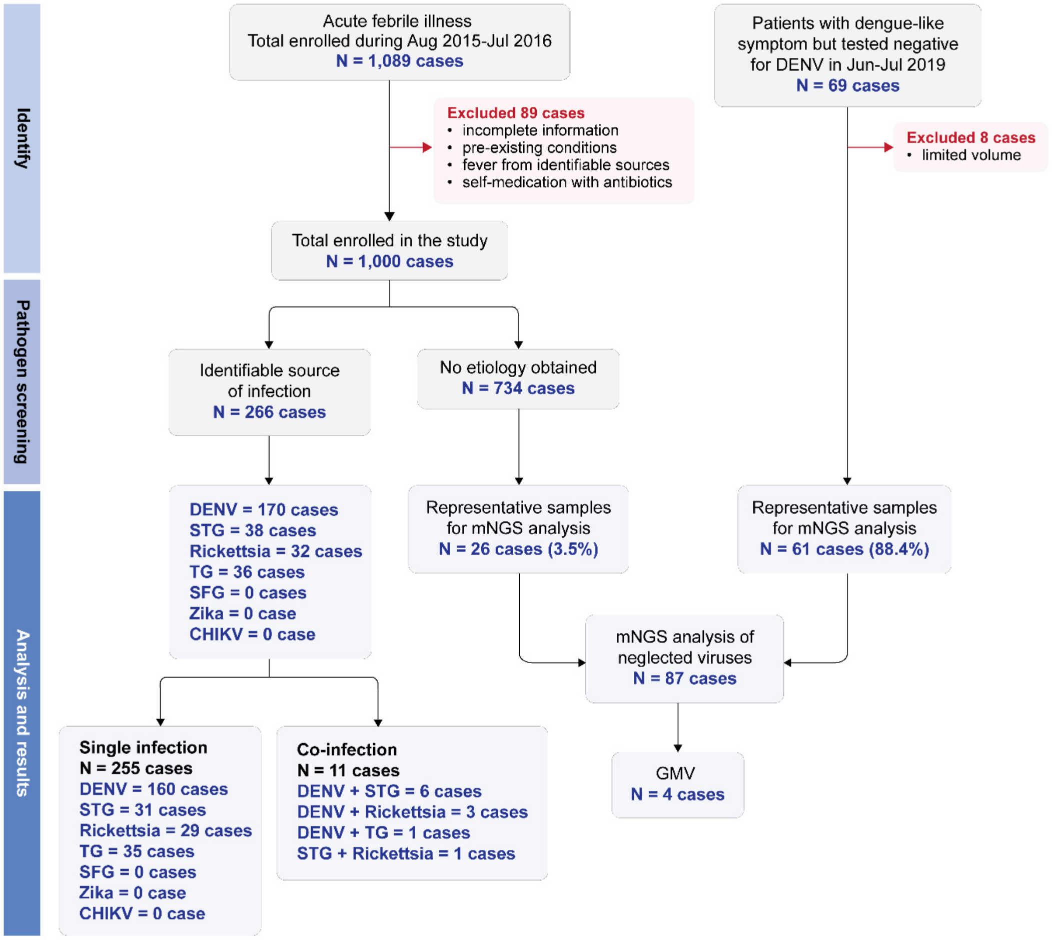

Figure S1. Sample selection flowchart.

Table S1. Designed primer sets for GMV detection and complete genome identification.

GMV	Fragment no.	Oligo name	Sequence (5′ to 3′)	Primer	Product size (bp)
RdRp region	1st PCR	GMV_RdRp_F1-1947	CTGGCTACTGTATTYTGACTC	Forward	420
	2nd PCR	GMV_RdRp_F2-2008	TATATGCTGTGATCCGGATAG	Forward	360
		T7-GMV_RdRp_R1-2370	TAATACGACTCACTATAGGGTGTACAGTC-CARTCCCAAGC	Reverse	
Genome segment 1		GMV_Seg1_F1-2	TTCCTAACTGTAAAACGAGATG	Forward	
	1_1st PCR	GMV_Seg1_R1-900	AAGGCGCTCCACTAAATCCT	Reverse	899
	1_2nd PCR	GMV_Seg1_nested R1-790	ACACTCAAGCCGGCATCTTT	Reverse	789
		GMV_Seg1_F2-703	GCAATCCAACACTGATAACGC	Forward	
	2_1st PCR	GMV_Seg1_R2-1466	ATAGGCGCCACAAGACTCAC	Reverse	764
	2_2nd PCR	GMV_Seg1_nested R2-1424	TTTACTCTTAGGTTCTGGAGGA	Reverse	722
		GMV_Seg1_F3-1252	TTGATCTCGAAGCTGTAACCA	Forward	
	3_1st PCR	GMV_Seg1_ R3-2230	TCTGGCTATCCAAGCGAACA	Reverse	979
	3_2nd PCR	GMV_Seg1_nested R3-2138	TCATTTTATGCGGCTCTTGCT	Reverse	887
	4_1st PCR	GMV_RdRp_F2-2008	TATATGCTGTGATCCGGATAG	Forward	1,031
	4_2nd PCR	GMV_Seg1_nested F4-2059	AAGGTTCAATGAGTTGTTGGC	Forward	980
		GMV_Seg1_R4-3038	ACTCATCCCGTTTCACTCAC	Reverse	
Genome segment 2		GMV_Seg2_F1-147	CACAATGAGTAAACAGTTGAAGA	Forward	
	1_1st PCR	GMV_Seg2_ R1-1003	CTGCATACGTTATCATATCCCT	Reverse	857
	1_2nd PCR	GMV_Seg2_ nested R1-902	GCCCGCACTTCATATAGGGT	Reverse	756
	2_1st PCR	GMV_Seg2_F2-770	ACATCTGGATCCACTATAAGG	Forward	893
	2_2nd PCR	GMV_Seg2_nested F2-839	CTCCAGGACCTAGTGTGTTT	Forward	824
		GMV_Seg2_R2-1662	TAACGACACGTTGGCAAACTA	Reverse	

Table S2. Information on the Renna virus and 17 GMV references used for specific-primer design in genome sequence study.

Genome	Accession numbers	Virus strain	Length (bp)
Segment 1	MK285337.1	Renna virus clone RENV_S1_Mex_2016	2,739
	MN053793.1	Guadeloupe mosquito virus strain Ab-AAM-5	3,136
	MN053803.1	Guadeloupe mosquito virus strain PB-AAM-1-4	3,119
	MN053801.1	Guadeloupe mosquito virus strain PB-AAM-5	3,174
	MN053797.1	Guadeloupe mosquito virus strain PB-AAM-1-2	3,127
	MN053805.1	Guadeloupe mosquito virus strain Ab-AAF	3,095
	MN053799.1	Guadeloupe mosquito virus strain PB-AAM-1-3	3,039
	MN053791.1	Guadeloupe mosquito virus strain Ab-AAF-5	3,142
	MN053807.1	Guadeloupe mosquito virus strain Ab-AAM	3,141
	MN053795.1	Guadeloupe mosquito virus strain PB-AAF-1-3	3,100
	MN053789.1	Guadeloupe mosquito virus strain Ab-AAF-1-5	3,084
	MN053787.1	Guadeloupe mosquito virus strain Ab-AAF-1-1	3,104
	MW434817.1	Guadeloupe mosquito virus isolate CMS002_017a_SAND	3,110
	MW434816.1	Guadeloupe mosquito virus isolate CMS002_018a_SAND	3,114
	MW434815.1	Guadeloupe mosquito virus isolate CMS002_018b_SAND	3,137
	MW434814.1	Guadeloupe mosquito virus isolate CMS002_017b_SAND	3,138
	MW434813.1	Guadeloupe mosquito virus isolate CMS002_017e_SAND	3,166
	MW434812.1	Guadeloupe mosquito virus isolate CMS002_019a_SAND	3,167
Segment 2	MK285338.1	Renna virus clone RENV_S2_Mex_2017, partial genome	1,131
	MN053794.1	Guadeloupe mosquito virus strain Ab-AAM-5	1,626
	MN053804.1	Guadeloupe mosquito virus strain PB-AAM-1-4	1,731
	MN053802.1	Guadeloupe mosquito virus strain PB-AAM-5	1,640
	MN053798.1	Guadeloupe mosquito virus strain PB-AAM-1-2	1,664
	MN053806.1	Guadeloupe mosquito virus strain Ab-AAF	1,628
	MN053800.1	Guadeloupe mosquito virus strain PB-AAM-1-3	1,615
	MN053792.1	Guadeloupe mosquito virus strain Ab-AAF-5	1,640
	MN053808.1	Guadeloupe mosquito virus strain Ab-AAM	1,590
	MN053796.1	Guadeloupe mosquito virus strain PB-AAF-1-3	1,604
	MN053790.1	Guadeloupe mosquito virus strain Ab-AAF-1-5	1,696
	MN053788.1	Guadeloupe mosquito virus strain Ab-AAF-1-1	1,683
	MW434811.1	Guadeloupe mosquito virus isolate CMS002_017a_SAND	1,586
	MW434805.1	Guadeloupe mosquito virus isolate CMS002_018a_SAND	1,633
	MW434810.1	Guadeloupe mosquito virus isolate CMS002_018b_SAND	1,597
	MW434806.1	Guadeloupe mosquito virus isolate CMS002_017b_SAND	1,583
	MW434809.1	Guadeloupe mosquito virus isolate CMS002_017e_SAND	1,619
	MW434804.1	Guadeloupe mosquito virus isolate CMS002_019a_SAND	1,637

Table S3. Sequencing read statistics and host filtering.

Sample pools	Raw reads	Filtered reads	% human-aligned reads
VF1	661,242	576,511	1.5%
VF2	617,803	529,469	5.4%
VF3	667,502	567,392	8.0%
VF4	774,779	675,115	11.4%
VF5	604,549	522,602	10.9%
VF6	851,898	732,086	2.9%
VF7	1,229,577	1,091,541	3.3%
VF8	726,914	593,816	4.3%
VF9	627,899	561,609	4.5%

Table S4. The normalized for million reads and BlastX analysis of each contig.

Sample pool	Contig name	Reads per million	Accession	Identity	*Name virus*
VF1	Contig701	487	QRW42396.1	51.43%	*Guadeloupe mosquito virus*
VF1	Contig26	27013	QEM39253.1	96.20%	*Guadeloupe mosquito virus*
VF2	Contig878	634	QEM39253.1	88.24%	*Guadeloupe mosquito virus*
VF2	Contig59	43433	QEM39253.1	100.00%	*Guadeloupe mosquito virus*
VF3	Contig101	44	QEM39257.1	82.98%	*Guadeloupe mosquito virus*
VF4	Contig1125	15	QEM39257.1	72.41%	*Guadeloupe mosquito virus*
VF4	Contig99	1298	QEM39273.1	100.00%	*Guadeloupe mosquito virus*
VF5	Contig1165	4883	QEM39257.1	81.63%	*Guadeloupe mosquito virus*
VF5	Contig35	13514	QEM39285.1	98.85%	*Guadeloupe mosquito virus*
VF6	Contig1146	15	QEM39253.1	92.59%	*Guadeloupe mosquito virus*
VF7	Contig10841	89	QEM39256.1	91.38%	*Guadeloupe mosquito virus*
VF8	Contig31	26762	QEM39285.1	100.00%	*Guadeloupe mosquito virus*
VF2	Contig28	26748	QBA55493.1	92.31%	*Renna virus*
VF2	Contig45	249668	QBA55493.1	97.97%	*Renna virus*
VF5	Contig663	428	QBA55493.1	100.00%	*Renna virus*
VF7	Contig2002	74	QBA55493.1	95.59%	*Renna virus*
VF9	Contig76	199256	YP_009448556.1	42.47%	*Orpheovirus IHUMI-LCC2*
VF3	Contig18	89198	UHK03708.1	99.64%	*Torque teno midi virus*
VF4	Contig1161	1077	UHK06663.1	70.51%	*Torque teno midi virus*
VF5	Contig19	752	UHK06680.1	100.00%	*Torque teno midi virus*
VF9	Contig640	15	UHK03708.1	96.63%	*Torque teno midi virus*
VF8	Contig2	73191	UGV44924.1	99.40%	*Torque teno virus*
VF9	Contig599	30	UGV44924.1	100.00%	*Torque teno virus*
VF9	Contig41	30	UGV44924.1	100.00%	*Torque teno virus*
VF5	Contig8	18500	UGV33890.1	96.67%	*TTV-like mini virus*
VF5	Contig4	29049	UGV36855.1	99.64%	*TTV-like mini virus*
VF8	Contig1	5665	UGV38306.1	97.95%	*TTV-like mini virus*
VF3	Contig121	47845	YP_003969769.1	25.49%	*Cafeteria roenbergensis virus*
VF6	Contig109	5385	AAB22972.1	98.58%	*Dengue virus 1*
VF9	Contig30	4057	UHS18432.1	57.14%	*Gammatorque virus sp.*
VF1	Contig340	2124	WET51920.1	76.19%	*Wenzhou sobemo-like virus 4*
VF9	Contig92	30	WET51920.1	97.22%	*Wenzhou sobemo-like virus 4*
VF3	Contig328	752	YP_667900.1	38.59%	*Neodiprion abietis NPV*
VF7	Contig1855	59	QOI91445.1	65.52%	*Kwale mosquito virus*
VF2	Contig402	44	QQN95304.1	91.89%	*Hubei mosquito virus 2*
VF7	Contig366	30	AAB66528.1	71.43%	*Multiple sclerosis associated retrovirus*
VF9	Contig7	66552	YP_009015548.1	31.71%	*Bacillus virus G*
VF8	Contig97	53362	YP_004306459.1	67.14%	*Burkholderia phage KL3*
VF8	Contig7	3260	YP_008766887.1	36.72%	*Shigella phage SfIV*
VF2	Contig13	2847	YP_009008153.1	49.21%	*Synechococcus phage ACG-2014h*
VF9	Contig22	988	YP_009147760.1	46.94%	*Lactococcus phage WRP3*
VF7	Contig3878	428	YP_762585.1	40.95%	*Lactococcus phage Q54*
VF7	Contig1103	221	YP_009616323.1	30.09%	*Rhodobacter virus RcCronus*
VF7	Contig7326	89	YP_004957306.1	34.62%	*Planktothrix phage PaV-LD*
VF7	Contig9719	30	YP_008531773.1	76.47%	*Propionibacterium virus PHL037M02*
VF7	Contig9718	15	YP_009159919.1	100.00%	*Propionibacterium virus Stormborn*
VF2	Contig914	15	YP_009497840.1	66.67%	*Escherichia virus G4*

Table S5. GMV patient information and hematology lab results.

Information	Normal value	GMV Patient code			
		S90	S247	D256*	D341*
**Age**		10 years	40 years	3.7 years	8.3 years
Gender		Male	Female	Female	Female
Body Temperature	<37.5	38.6	38.4	38.5	39.7
Fever period before admission		1 day	1 day	4 days	4 days
Occupation		Under the guardianship of parents	Contract	Under the guardianship of parents	Under the guardianship of parents
**Hematology lab**					
WBC	4,500-10,000 Cells/µL	↑16,700	↑26,300	4,600 (↓4,200-5,000)	5,650 (↓3,600-↑11,700)
Red blood cell	$4.2x10^6$-$6.3x10^6$ Cells/µL	$5.1x10^6$	$5.1x10^6$	$5.4x10^6$ (5.1-$5.6x10^6$)	$5.8x10^6$ (4.8-$6.5x10^6$)
Hemoglobin	M:13-17.4, F:12-16 g/dL	13.4	↓8.7	12.9 (12.1-13.2)	14 (↓11.6-15.4)
Hematocrit	M:40-54, F:37-47 %	↓37.9	↓28	38.5 (↓36.8-40.1)	41.7 (↓36.3-46.3)
MCV	80-100 fL	↓74	↓55	↓72 (↓71-↓72)	↓72.5 (↓72-↓75)
MCH	27-32 pg	↓26.1	↓17.2	23.8 (23.5-24.1)	24.1 (23.6-24.3)
MCHC	32-36 g/dL	35.2	↓31.2	33.2 (32.8-33.5)	33 (32-33.5)
RDW	11.5-14.5 %	12.6	↑17.2	↑15 (14.4-↑15.7)	↑15 (13.6-↑16.3)
Platelet count	140,000-400,000 Cells/µL	352,000	352,000	148,500 (↓121,000-234,000)	↓98,000 (↓29,000-357,000)
Neutrophil	50-70 %	63.4	↑89	↓46.8 (↓38.1-↓48.3)	58.3 (↓17-↑81.6)
Lymphocyte	20-40 %	25.9	↓5.5	43.85 (42.6-↑52.4)	36.45 (↓10.6-↑68)
Monocyte	2-8 %	↑8.8	4.1	↑8.1 (6.6-↑8.5)	6.1 (3.3-↑11.2)
Eosinophil	1-6 %	1.3	1.1	↓0.7 (↓0.3-↓0.8)	↓0.6 (↓0-1.2)
Basophil	0-1 %	0.6	0.3	0.8 (0.5-↑1.5)	0.4 (0-0.5)
Atypical lymp	0-2.5 %	1	0.5	1 (0.8-1.5)	1.1 (0.7-↑9)

Note: WBC = White blood cell Count, MCV = Mean corpuscular volume, MCH = Mean corpuscular hemoglobin, MCHC = Mean corpuscular hemoglobin concentration and RDW = Red blood cell distribution width. * = Median (min-max) for hematology laboratory results.
